# Evaluation of six serological ELISA kits available in Italy as screening tests for equine infectious anaemia surveillance

**DOI:** 10.1186/s12917-017-1007-6

**Published:** 2017-04-14

**Authors:** Roberto Nardini, Gian Luca Autorino, Charles J. Issel, R. Frank Cook, Ida Ricci, Raffaele Frontoso, Francesca Rosone, Maria Teresa Scicluna

**Affiliations:** 1 Istituto Zooprofilattico Sperimentale del Lazio e della Toscana “M. Aleandri”, Via Appia Nuova 1411, 00178 Rome, Italy; 2 Department of Veterinary Science, Gluck Equine Research Center, University of Kentucky, Lexington, KY USA

**Keywords:** Equine infectious anaemia, ELISA, AGID, Comparison, Commercial assays, In-house assays, Surveillance

## Abstract

**Background:**

ELISAs are known to have a higher diagnostic sensitivity than the agar gel immunodiffusion (AGID) when employed for serological diagnosis of equine infectious anaemia (EIA). For this purpose, an “in-house” and five commercial ELISAs available in Italy were assessed by the National Reference Centre for EIA for their analytic specificity (Sp); precocity, defined as capability of detecting first antibodies produced during a new infection; precision based on repeatability and reproducibility, estimated from the coefficient of variation (CV); accuracy, estimated from multiple K and relative Sp and sensitivity (Se). Two serum panels, positive for non-equine retroviruses and the most frequent equine viruses, were employed to measure analytic Sp. ELISA precocity was also compared to that of one “in-house” and three commercial AGID kits, employing a panel of sera, collected weekly from horses infected with modified EIA viruses. Precision and accuracy were defined using results of a panel containing positive and negative sera examined in an inter-laboratory trial with the participation of the ten Official Laboratories. Furthermore, a questionnaire was used to assess the appropriateness of each kit for routine use.

**Results:**

Analytic Sp was 100%, while the 75th percentile of CVs for positive sera varied from 0.4% to 12.73% for repeatability and from 1.6% to 44.87% for reproducibility. Although CV of the negative serum was constantly high, its outcome was unaltered. Relative Se ranged from 98.2% to 100%, relative Sp was constantly 100% and multiple K ranged from 0.95 to 1. Precocity differed among the assays: three kits detected 4.8% and 42.9% positive samples on 21 days post infection (dpi), all assays detected positive samples on 28 dpi, between 47.6% and 95.2%. Precocity of ELISAs was superior to that of the AGIDs except for two assays. In view of the feedback obtained from the questionnaires, all kits were considered appropriate for routine use.

**Conclusion:**

All ELISAs having high Se and precocity are preferable as a screening test in EIA surveillance programmes to the AGID tests examined. These two tests can be incorporated in a serial diagnostic pathway to improve the efficacy of a surveillance plan.

## Background

EIA is a blood-borne disease of equids caused by EIA virus (EIAV) of the Retroviridae family, subfamily Orthoretrovirinae, genus Lentivirus. EIA has a nearly worldwide distribution and causes sanitary and economic repercussions on the horse industry. In view of this, it is among the eleven equine diseases listed by the World Organisation for Animal Health (OIE). In addition, in several countries it is included in national and international Regulations defining the sanitary conditions for horse trade and movement and is also subjected to regional or national control programmes.

EIAV detection is generally problematical [1, 2], hence diagnosis of suspect cases and screening during surveillance programmes are based on antibody detection [3]. At present, for surveillance purposes, the OIE considers ELISA as a secondary method to the AGID, reporting that various factors limit its use. Nevertheless, it is also indicated as suitable for the declaration of population freedom from disease and for the evaluation of the efficiency of eradication programmes. The OIE also recommends that samples positive in ELISA are to be confirmed using either the AGID or the immunoblot (IB) [4]. These three methods have been proposed in a three-tier system that increases the diagnostic efficacy of EIA [5].

Since 2007, a surveillance programme for the identification and removal of EIA positive animals is on-going in Italy. In its initial phases, the AGID was employed both as screening and as confirmatory method respectively by the network of the ten Official Laboratories and the National Reference Centre (NRC) for EIA. In recent years, scientific reports [6–8], including those from Canada [9] and the USA [5], have demonstrated that ELISA-based tests are potentially more sensitive than AGID. The NRC adopted a similar approach to that described in these studies to evaluate, on the basis of their key diagnostic characteristics, the possibility of employing ELISA kits available in Italy for the serological screening of equid serum samples examined within the National surveillance programme. The results obtained, including a comparison with four different AGID kits, are presented and discussed especially in terms of the implications of the use of the ELISAs on the efficacy of the EIA surveillance.

## Methods

A summary of the technical characteristics of the ELISAs employed in the present study are presented in Table 1. One ELISA is an “in-house” assay while the remaining are commercially produced. Following invitation to participate to the present evaluation, each producer kindly provided a kit of the batch that was at that time available on the market. ELISA #1 [10] and #2 [Scicluna, personal communication, 2016] were the kits that at the time of the study were already validated according to the OIE guidelines [11]. All the immunoassays were performed according to the manufacturer’s instructions.

The AGID assays included in the comparison analysis were performed according to the OIE procedure [4] and consisted in one in-house and three commercially available AGIDs. Two AGIDs employed a recombinant EIAV p26 antigen, the third reported the use of a highly purified recombinant viral protein without providing further information and the remaining kit did not include details relative to the source of the antigen employed.

The composition and characteristics of the four panels of sera employed in the evaluation of the ELISAs are described in Table 2 and these will be referred throughout by an identification number accompanied by a brief description.

The parameters by which the ELISAs were assessed are: analytic Sp that was defined employing serum samples containing antibodies to non-equine retroviruses (Panel 1), along with a set of sera, positive for the most common equine viral diseases (Panel 2) [12]. Each serum sample included within these panels was examined in 30 replicates.

Precocity of both ELISA and AGID tests, defined as the capability to detect the first amounts of antibodies produced during the onset of an infection, was assessed using equine serum samples collected at weekly intervals (day 0 to 28 dpi), from 3 groups of eight horses infected with one of the following EIAV attenuated strains [10]: EIAV_D9_, EIAV_Trivalent_ and EIAV_Consensus_. Three of the 24 horses, two infected with EIAV_Consensus_ and one with EIAV_D9,_ seroconverted after 28 dpi (Issel CJ, personal communication, 2014), therefore for this study only the samples of the 21 positive horses were included in Panel 3.

For the statistical analysis, the Chi Square test was used to compare the proportions of reactive samples (positive and equivocal) detected by each method and in case of a statistical significant difference, the Marascuilo procedure was employed to investigate for which pair of assays this occurred. Samples with an equivocal result to the screening test were reclassified as positive since these, as for the positively reacting samples, are also forwarded to the NRC for confirmation.

XLStat (Addinsoft®, 2011) was the software utilized for the statistical analysis and a test obtaining a *p* value ≤0.05 was considered significant.

The results obtained for panel 4, used in the inter-laboratory trial (IT) to which the ten Official Laboratories participated [13], were analysed to assess precision and accuracy. The panel was examined blindly and consisted of one negative reference serum, included as eight replicates, and 11 positive samples each provided as two replicates. The positive samples were prepared from one positive reference serum diluted with a negative one to obtain three different reactivity levels that were arbitrarily defined as follows by the NRC, on the basis of the percentage inhibition (PI) obtained in ELISA #1: strong positive (SP) with a PI ≥95% (*n* = 2), medium positive (MP) with a PI between 75% and 94% (*n* = 3) and weak positive (WP) with a PI between 51% and 74% (*n* = 6). The positive sera were also confirmed, except for one WP, in the in-house AGID described above. Both in-house tests, in use at the NRC, are accredited according to ISO/IEC 17025 [14].

Precision was assessed using repeatability and reproducibility, which were calculated as follows. Repeatability was measured using the CV of the results of the two replicates of each positive serum and of the eight replicates of the negative obtained in each ELISA run by every IT participant. The CVs were calculated using the percentage inhibition (PI) or corrected sample/positive ratio (S/P) values (see formulae in Table 1). For those assays without an interpretation formula, the results were normalized according to their set-up, using either the PI (ELISA #3) or the S/P (ELISA #5 and ELISA #6).

**Table 1:** Principal characteristics of the ELISAs employed in the study

	ELISA #1	ELISA #2	ELISA #3	ELISA #4	ELISA #5	ELISA #6
Type	Competitive	Indirect	Competitive	Competitive	Indirect	Indirect
Antigen	Recombinant p26	Recombinant *gag* and *env* antigens	Purified p26	Recombinant p26	Recombinant antigen- not specified	Recombinant p26
Run time (‘)	160	135	45	90	20	35
Interpretation Criterion/Formula	$\mathrm{PI}=100-\left({\overline{\mathrm{OD}}}_{\mathrm{S}}/\overline{{\mathrm{OD}}_{\mathrm{K}-}}\ast 100\right)$	$\mathrm{S}/\mathrm{P}={\frac{\left(\overline{{\mathrm{OD}}_{\mathrm{S}}}-\overline{{\mathrm{OD}}_{\mathrm{K}-}}\right)}{\left(\overline{{\mathrm{OD}}_{\mathrm{K}+}}-\overline{{\mathrm{OD}}_{\mathrm{K}-}}\right)}}^{\ast}\;100$	Compared to OD_K+_	$\mathrm{S}/\mathrm{P}={\frac{\left(\overline{{\mathrm{OD}}_{\mathrm{S}}}-\overline{{\mathrm{OD}}_{\mathrm{K}-}}\right)}{\left(\overline{{\mathrm{OD}}_{\mathrm{K}+}}-\overline{{\mathrm{OD}}_{\mathrm{K}-}}\right)}}^{\ast}\;100$	Compared to OD_K+_	Compared to OD_K+_
	PI <30: Neg	S/P ≤ 40: Neg	OD_S_ > OD_K+_: Neg	S/P ≤ 50: Neg	OD_S_ < OD_K+_: Neg	OD_S_ < OD_K+_: Neg
Outcome definition	30 ≤ PI ≤ 50: Equivocal	40 < S/P < 50: Equivocal	OD_S_≤ OD_K+_: Pos	50 < S/P < 60: Equivocal	OD_S_ ≥ OD_K+_: Pos	OD_S_ ≥ OD_K+_: Pos
	PI >50: Pos	S/P ≥ 50: Pos		S/P ≥ 60: Pos		

*Gag* and *env:* genes of EIAV respectively coding for nucleocapsid and envelope antigens, PI: percentage inhibition, S/P: Corrected Sample /Positive Ratio;

OD_S_: Optical Density of Sample; OD_K-_: Optical Density of Negative Control; OD_K+_: Optical Density of Positive Control; Neg: Negative; Pos: Positive

**Table 2:** Characteristics of the panels of sera employed in the study

Panel #	Characteristics
1: Sera positive for non-equine retroviruses	20 sera for feline immunodeficiency virus
	20 for feline leukemia virus
	20 for enzootic bovine leukosis virus
	20 for maedi visna virus
2: Sera positive for other equine viruses	20 sera positive for equine influenza
	20 sera positive for equine viral arteritis
	20 positive for equine herpes virus −1
	20 sera positive for equine herpes virus −4
3: Sera of 21 horses experimentally infected^b,c^	84 sera sampled at 0, 14, 21, 28 days post infection (dpi)
4: Interlaboratory test panel^a^	30 sera: 22 positive and 8 negative

^a^Available at the EIA National Reference Centre and at the National Reference Centre for Equine Diseases as secondary reference sera

^b^Provided by Gluck Equine Research Center University of Kentucky Lexington, Kentucky

^c^Infected with EIA attenuated virus EIAV^D9^ EIAV^Trivalent^, EIAV^Consensus^ (further information is provided in the text)

**Table 3:** Values of accuracy, multiple K, and total scores obtained from the questionnaire

ELISA	Re_Se_ (%)	Re_Sp_ (%)	Multiple K	Score (maximum score 65)
#1	99.5	100	0.99	56
#2	99.1	100	0.98	57
#3	98.2	100	0.95	59
#4	99.1	100	0.98	63
#5	96.8	100	0.96	58
#6	100	100	1	60

Re_Se_: relative sensitivity; Re_Sp_: relative specificity

Reproducibility of the assays was evaluated using the CVs estimated from the gathered results of the IT.

The accuracy of the ELISA kits was defined from the values obtained for multiple K and relative Sp and Se. The latter were calculated as the percentage of samples identified over the total number of expected results, respectively for the negative and positive sera, independently from the different reactivity level. For the reason mentioned previously, sera with equivocal results were also here reclassified as positive.

Concordance with the expected results was estimated, for each laboratory, using K coefficient [15] for those assays (ELISA #3, #5 and #6) with two possible outcomes (positive and negative) and with a modified K coefficient, i.e. weighted K [16] for those ELISAs (#1, #2 and #4) with three expected outcomes: positive, equivocal and negative, arbitrarily assigning a weight of 0.33 to a partial concordance. The results of all the laboratories were then aggregated to calculate the multiple K value [17] of each assay, which was interpreted as reported in literature [18].

Furthermore, the laboratory personnel participating in the IT also replied to a questionnaire to investigate the suitability of the ELISA kits for routine use. This survey contained 17 questions covering 15 criteria, with preset answers represented by a scale of 1 (lowest rating) to 5 (highest rating). The questions focused on ease of interpretation of procedures, reagents characteristics, ease and time of execution, straightforwardness of result formula and result interpretation. Overall rating for each question was assigned using the mode obtained from all the scores reported by the laboratories and summed for each kit. In addition, the questionnaire also included two open questions requesting advice and recommendations for improvements of each kit.

## Results

**Fig. 1:**
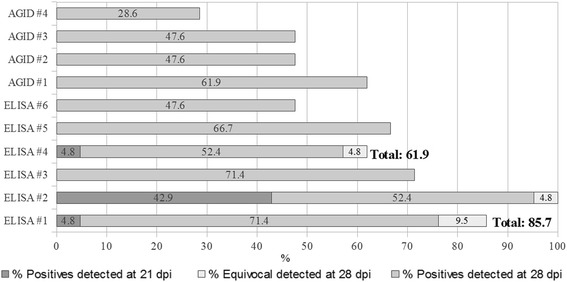
Percentage of positive and equivocal samples detected by each ELISA and AGID assay for sera sampled at 21, 28 dpi with attenuated EIAV strains (EIAV_D9_, EIAV_Trivalent_ and EIAV_Consensus_)

Analytical Sp was 100% for all ELISAs as no cross-reactivity was detected for sera of Panel 1 (positive for non-equine retroviruses) and Panel 2 (positive for common equine diseases). Data on the analytical Se is presented in Fig. 1 and was assessed by the precocity of antibody detection in the horse sera (Panel 3) collected at weekly intervals following infection with modified live viruses. The results of this parameter are expressed as the percentage of the number of samples detected positive/equivocal out of the total samples expected as positive. Positive samples were detected from 21 dpi, in ELISA #1 and ELISA#4 identifying each a different positive sample (4.8%) and ELISA #2, identifying 9 positive samples (42.9%), including the two detected by ELISAs #1 and #4; on 28 dpi all assays detected a percentage of positive/equivocal samples, ranging from 47.6% (ELISA #6) to 100% (ELISA #2).

Positive samples were also detected at 28 dpi by the different AGID assays with percentages ranging between 28.6% (AGID #4) and 61.9% (AGID #1). In comparing the precocity of the two different methods, for ELISA #6 this was equal (47.6%) to those of AGIDs #2 and #3 and for ELISA #4 it was equal (61.9%) to that of AGID #1 (Fig.1).

No statistical differences were observed among the AGID kits in terms of percentage of positive/equivocal samples detected. For the ELISAs, differences were detected at 21 dpi with a *p* value of <0.0001 for the Chi Square test and with the Marascuilo procedure showing a statistical difference between the proportions of positives detected by ELISA #2 with those of ELISAs #3, #5 and #6. Even on 28 dpi, the *p* value was statistically significant with a value of 0.018 and differences were detected between the proportions of ELISA #2 and ELISAs #4 and #6.

**Fig. 2:**
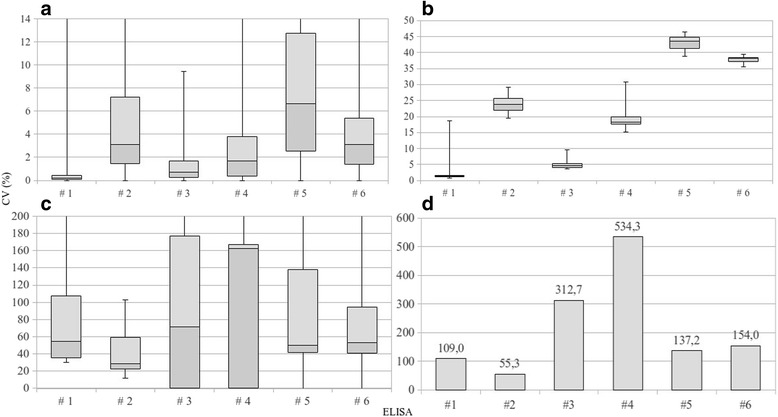
Box plot graphs of CVs of repeatability and reproducibility, respectively for positive (**a**, **b**) and negative (**c**, **d**) sera for each assay. Lower box represents difference between first quartile and median and upper box difference, between third quartile and median. Y bars represent minimum and maximum values (due to the graphical representation some values are out of scale). Graph D represents the CVs of the negative serum considering all laboratories

The distribution of the CV values for repeatability and reproducibility for each of the assays and for all laboratories are shown in Fig. 2. The 75th percentile of the CVs of the positive sera did not exceed 12.73% for repeatability (Fig. 2a) and 44.87% for reproducibility (Fig. 2b). Conversely, for the negative serum, CVs at the 75th percentile were high with a maximum of 137.54% for repeatability (Fig. 2c) and 534.31% (Fig. 2d) for reproducibility, without ever modifying the expected outcome.

The results of the accuracy and total scores obtained from the questionnaires are reported in Table 3. Relative Sp was 100% for all the ELISAs while relative Se ranged from 96.8% (ELISA #5) to 100% (ELISA #6), with all the remaining kits having a relative Se of >98%. Three laboratories misclassified sixteen sera represented by 1 SP, 2 MP and 13 WP (7 of which corresponded to that negative in the AGID).

Values of Multiple K ranged from 0.95 (ELISA #3) to 1 (ELISA #6), resulting in an “almost perfect” concordance according to the adopted classification. Scores for kit quality perception were very high for all the ELISAs and varied from 56 (ELISA #1) to 63 (ELISA #4) on a maximum score of 65.

Comments provided by laboratory participants were reporting as critical the small sample volume used in ELISA #2, the absence of hazard and safety statements for kit reagents in ELISAs #3, #4 and #5, the quality of the reagent containers used in ELISAs #1 and #2, as they were prone to leaking, and the orientation of the well-strips of ELISA #3. In contrast to all other assay formats, the individual strips of this kit were made up of twelve wells instead of the more often available format of eight wells and therefore considered impractical when used in preset instruments such as automated washers.

## Discussion

Results of the present study demonstrated that, except for precocity, the ELISA kits currently available in Italy for serological diagnosis of EIA offered similar performance levels. All exhibited high analytical Sp (100%) and accuracy, with a relative Se between 98.2% and 100%, a relative Sp of 100% and multiple K values ranging from 0.95 to 1 (see Table 3). Sera misclassification mostly occurred for the WP (81.3%), with 43.8% of these corresponding to the WP-AGID negative serum. A misclassified sample could be due either to the test sensitivity or to the laboratory’s technical capability. In particular, the WP-AGID negative sample was detected as negative by 3 laboratories in one or more of the following ELISAs: #1, #2, #3, and #5. For the latter two ELISAs, misclassification could be related to their low precocity. Differently for the other two kits, which demonstrated a high precocity, the use of relatively small serum volumes could influence their sensitivity, especially when using uncalibrated instrumentation.

All assays were sufficiently repeatable and reproducible as CV values obtained for the positive sera are similar to those described in the literature [19–21]. The higher magnitude of the CVs observed for the negative serum is due to the low values that this assumes when using the PI and S/P formulae. In this case, small numerical differences have a greater weight when calculating the CV [9].

Screening tests in a surveillance programme should detect all levels of true positivity, especially those at borderline level, as a false negative serum would immediately be lost and consequently diminish the efficacy of the control programme.

Sensitivity, which in this study was also assessed through precocity, is essential for avoiding the persistence of EIA, not only through the early detection of new cases that are still mounting their antibody response but also by the identification of animals with a low serological response [8] that may occur during the chronic or inapparent phases of AIE and which was evaluated by the inclusion of WP sera in the IT panel. The best performing kit for precocity was ELISA #2, which could be ascribed to the use of *env* gene-derived recombinant antigen in combination with the p26, employed by all the other assays [22]. Previous studies report that antibodies against the glycoproteins (transmembrane -gp45 and surface unit -gp90) expressed by the *env*-gene are produced earlier than those against the p26 [23–26]. Lacking complete information on the immunological reagents employed in the ELISAs, this hypothesis requires further investigation, considering also that a previous study describes that an assay containing just the gp45 had a lower performance than those using the p26 [9].

Further investigations are also required to verify the data obtained for ELISAs #4 and #6, as their precocity did not exceed that of some of the AGIDs. However, the performance of these assays relative to the other parameters was perceived as good with relative Sp and Se values equal to 100% for the first and respectively 99.1% and 100% for the second, while those of multiple K were 1 for ELISA #4 and 0.98 for ELISA #6. The evaluation of ELISA kits described here, together with results reported by other authors [5–9], confirm that most of these test-formats have higher or equal levels of overall sensitivity than the AGID assay, even if this method is still considered as the “Gold Standard” by many governmental regulatory authorities and is also indicated as an appropriate screening test by the OIE [4]. In addition to the present data, a limit of detection (LOD) for the AGID of at least 0.9 Log_10_ lower than that of the ELISA was already reported by the same authors [10]. Laboratories employing this method as screening test are strongly advised to replace it with a more sensitive ELISA to increase the benefits of their control programmes.

The approach presented here for the evaluation of the performance of the ELISA kits provides the technical basis for establishing the criteria of choice of a diagnostic assay within the context in which it is be employed. However, further to the evaluation of the diagnostic parameters that rely on laboratory data, the ease of use of a kit in the routine is also critical in determining its choice. For this, questionnaires are valid tools [27, 28] for considering aspects that are not directly dependant on the assay’s diagnostic performance, such as the availability and completeness of the validation data, clarity of the manufacturer’s instructions and practicability of execution.

The implications of the questionnaire go even beyond the purpose of this study, as the recommendations provided through these means are also useful to the manufactures. Worthy of note are the replies provided in the open questions section, relative to the 5 μl sample volume analysed by ELISA #2, which was judged as too small and perceived as a source of variability, even if this assay was highly performing in all aspects and especially for precocity. Further to what previously discussed, a cost benefit analysis should also be included in the decisional process for the choice of a kit, especially in terms of time of execution in view of the high turnover required in surveillance programmes where a large number of samples are generally examined.

The validation of diagnostic kits, based on the criteria included in international guidelines, such as those indicated by the OIE [29], provides essential information for their comparison. Such an approach is adopted in some countries, as prior to the first release of the diagnostic kits on the market official laboratories either directly validate the kit or verify the technical data provided by the manufactures. Further to this, every released batch has to be approved by the appointed laboratory through the verification of the performance of some of the key diagnostic parameters or from the data presented by the producer.

Validation, according to the international guidelines for those ELISAs (#3 to #6) which at the time of the present study was unavailable, would have required a consistent amount of resources, especially in terms of time and costs. For this reason, only parameters considered as critical were evaluated for the assessment of the diagnostic performance of the assays, especially those that define the kits’ sensitivity, fundamental characteristic for a screening test. The advantage of the simultaneous comparison of the ELISAs kits was that the sera panels used were the same for all of them, differently from what would have been the case in an independent evaluation, therefore increasing the objectivity of the data produced. In addition, it also gave the opportunity of using an assessment questionnaire that represents an innovative method of evaluation. Also, on completion of this study, a final report was provided to each manufacturer participating to this study, offering them the opportunity of an independent performance appraisal in relation to the other kits available on the market.

As the results of the present study are exclusively related to the batches that were provided at that time by the manufactures, the NRC established a set of verification criteria for monitoring batch-to-batch variability based on the results obtained in this study. The verification parameters consist in the comparison of the LOD of the ELISA to that of AGID, estimation of specificity and CV respectively assessed from a statistically representative number of certified negative and positive samples. The batches of the kits that are approved are then published on the NRC website so that the Official Laboratories may procure any one of them.

In view of the parameters evaluated, all the ELISAs had an equal analytic Sp of 100%, while the best performing kits were ELISA#2 for precocity, ELISAs #1 and #2, respectively for repeatability and reproducibility, which were a measure of precision, and ELISA #1 and ELISA #4 for accuracy.

## Conclusions

The present study demonstrates that in general, the six ELISAs available in Italy have similar performance characteristics except for precocity. In view of the points discussed, the EIA Italian surveillance programme adopted this method as screening test, instead of the former AGID based-system, to guarantee a major efficacy of the on-going surveillance programme. The results presented corroborate those obtained in similar studies and provide evidence to national and international regulatory bodies, including the OIE, to reconsider the use of high performing ELISAs for the estimation of prevalence infection and also as screening method for surveillance purposes. The other advantages of the use of this test to the AGID, are its standardization, automation, high turnover of samples, cost per determination, and objective reading of sample outcomes [10].

Also, according to the different Sp and Se characterizing the kits, the authors support the parallel use of different ELISAs, already proposed in the “multitier system” [5], which would enhance the sensitivity of the diagnostic system and, as reported in a previous study conducted by the authors, would require that only 1 ‰ of the examined samples be subsequently submitted to a higher level of diagnosis in IB [5].

Further to the diagnostic quality of the ELISAs, the technical capability of the laboratory network conducting the serological surveillance of EIA employing different tests should also undergo verification. This aspect, together with the certification of kits for their use within a diagnostic system, is among the institutional duties of a NRC. In view of this, the official laboratories are periodically evaluated through the organization of ITs where the sensitivity limits of the diagnostic system that is subject to verification are set through the inclusion of weak positive and/or equivocal sera.

This double aspect, made up of high performing laboratories and methods, guarantees the efficiency of a surveillance system.

## Abbreviations

AGID: Agar gel immunodiffusion
CV: Coefficient of variation
Dpi: Days post infection
EIA: Equine infectious anaemia
EIAV: Equine infectious anaemia virus
IB: Immunoblot
IT: Inter-laboratory trial
LOD: Limit of detection
MP: Medium positive
NRC: National Reference Centre
OIE: World Organisation for Animal Health
PI: Percentage inhibition
Re_Se_: Relative sensitivity
Re_Sp_: Relative specificity
S/P: Corrected sample/positive ratio
Se: Sensitivity
Sp: Specificity
SP: Strong positive
WP: Weak positive

## References

[1] Hines R, Maury W (2001). DH82 cells: a macrophage cell line for the replication and study of equine infectious anemia virus. J Virol Methods.

[2] Leroux C, Cadoré J-L, Montelaro R (2004). Equine Infectious Anemia Virus (EIAV): what has HIV’s country cousin got to tell us? Caroline. Vet Res.

[3] Issel CJ, Cook SJ, Cook RF, Cordes TR (1999). Optimal paradigms to detect reservoirs of equine infectious anemia virus (EIAV). J Equine Vet Sci.

[4] World Organization For Animal Health: Manual of Diagnostic Tests and Vaccines for Terrestrial Animals 2015. www.oie.int/fileadmin/Home/eng/Health_standards/tahm/2.05.06_EIA.pdf. Accessed 12 Feb 2016.

[5] Issel CJ, Scicluna MT, Cook SJ, Cook RF, Caprioli A, Ricci I, Rosone F, Craigo JK, Montelaro RC, Autorino GL (2013). Challenges and proposed solutions for more accurate serological diagnosis of equine infectious anaemia. Vet Rec.

[6] Lew AM, Thomas LM, Huntington PJ (1993). A comparison of ELISA, FAST-ELISA and gel diffusion tests for detecting antibody to equine infectious anaemia virus. Vet Microbiol.

[7] Matsushita T, Hesterberg LK, Porter JP, Smith BJ, Newman LE (1989). Comparison of diagnostic tests for the detection of equine infectious anemia antibody. J Vet Diagn Investig.

[8] Scicluna MT, Issel CJ, Cook FR, Manna G, Cersini A, Rosone F, Frontoso R, Caprioli A, Antognetti V, Autorino GL (2013). Is a diagnostic system based exclusively on agar gel immunodiffusion adequate for controlling the spread of equine infectious anaemia?. Vet Microbiol.

[9] Paré J, Simard C (2004). Comparison of commercial enzyme-linked immunosorbent assays and agar gel immunodiffusion tests for the serodiagnosis of equine infectious anemia. Can J Vet Res.

[10] Nardini R, Autorino GL, Ricci I, Frontoso R, Rosone F, Simula M, Scicluna MT (2016). Validation according to OIE criteria of a serologic competitive enzyme-linked immunosorbent assay as screening method in surveillance programs for the detection of Equine infectious anemia virus antibodies. J Vet Diagnostic Invest.

[11] World Organization for Animal Health (OIE) (2010). Manual of Diagnostic Tests and Vaccines for Terrestrial Animals, Chapter 1.1.5.

[12] Slater J (2014). Findings from the National Equine Health Survey, 2013. Vet Rec.

[13] Langton SD, Chevennement R, Nagelkerke N, Lombard B (2002). Analysing collaborative trials for qualitative microbiological methods: Accordance and concordance. Int J Food Microbiol.

[14] ISO/IEC 17025 General requirements for the competence of testing and calibration laboratories. International Organization for Standardization, 2005.

[15] Cohen J (1960). A Coefficient of Agreement for Nominal Scales. Educ Psychol Meas.

[16] Cohen J (1968). Weighted kappa: nominal scale agreement with provision for scaled disagreement or partial credit. Psychol Bull.

[17] Fleiss JL (1971). Measuring nominal scale agreement among many raters. Psycological Bull.

[18] Landis JR, Koch GG (1977). The measurement of observer agreement for categorical data. Biometrics.

[19] Chung C, Wilson C, Timoney P, Adams E, Adams DS, Chung JS, Evermann JF, Shuck K, Lee SS, McGuire TC (2013). Validation of an improved competitive enzyme-linked immunosorbent assay to detect Equine arteritis virus antibody. J Vet Diagn Investig.

[20] Colling A, Morrissy C, Barr J, Meehan G, Wright L, Goff W, Gleeson LJ, van der Heide B, Riddell S, Yu M, Eagles D, Lunt R, Khounsy S, Than Long N, Phong Vu P, Than Phuong N, Tung N, Linchongsubongkoch W, Hammond J, Johnson M, Johnson W, Unger H, Daniels P, Crowther JR (2014). Development and validation of a 3ABC antibody ELISA in Australia for foot and mouth disease. Aust Vet J.

[21] Paweska JT (2007). Jansen van Vuren P, Swanepoel R: Validation of an indirect ELISA based on a recombinant nucleocapsid protein of Rift Valley fever virus for the detection of IgG antibody in humans. J Virol Methods.

[22] Issel CJ, Cook RF, Mealey RH, Horohov DW: Equine infectious anemia in 2014: live with it or eradicate it? Vet Clin North Am Equine Pract 2014, 30:561–577. 10.1016/j.cveq.2014.08.002 25441114

[23] Ball JM, Henry NL, Montelaro RC, Newman MJ (1994). A versatile synthetic peptide-based ELISA for identifying antibody epitopes. J Immunol Methods.

[24] Carpenter S, Alexandersen S, Long MJ, Perryman S, Chesebro B (1991). Identification of a Hypervariable Region in the Long Terminal Repeat of Equine Infectious Anemia Virus. J Virol.

[25] Chong YH, Ball JM, Issel CJ, Montelaro RC, Rushlow KE (1991). Analysis of equine humoral immune responses to the transmembrane envelope glycoprotein (gp45) of equine infectious anemia virus. J Virol.

[26] Hussain KA, Issel CJ, Schnorr KL, Rwambo PM, West M, Montelaro RC (1988). Antigenic mapping of the envelope proteins of equine infectious anemia virus: identification of a neutralization domain and a conserved region on glycoprotein 90. Arch Virol.

[27] Crowther JR, Unger H, Viljoen GJ (2006). Aspects of kit validation for tests used for the diagnosis and surveillance of livestock diseases: producer and end-user responsibilities. Rev Sci Tech.

[28] Johnson AM, Roberts H, Tenter AM (1992). Evaluation of a recombinant antigen ELISA for the diagnosis of acute toxoplasmosis and comparison with traditional antigen ELISAs. J Med Microbiol.

[29] World Organization For Animal Health: Manual of Diagnostic Tests and Vaccines for Terrestrial Animals 2015. www.oie.int/fileadmin/Home/eng/Health_standards/tahm/1.01.06_VALIDATION.pdf. Accessed 12 Feb 2016.

